# Chronic Ly49H Receptor Engagement *in vivo* Decreases NK Cell Response to Stimulation Through ITAM-Dependent and Independent Pathways Both *in vitro* and *in vivo*

**DOI:** 10.3389/fimmu.2019.01692

**Published:** 2019-07-23

**Authors:** Phillip N. Key, Joe Germino, Liping Yang, Sytse J. Piersma, Sandeep K. Tripathy

**Affiliations:** ^1^Gastroenterology Division, Department of Medicine, Washington University School of Medicine, St. Louis, MO, United States; ^2^Rheumatology Division, Department of Medicine, Washington University School of Medicine, St. Louis, MO, United States

**Keywords:** NK cell, tolerance, activating receptor, signal transduction, ITAM, Ly49H, m157

## Abstract

Natural killer (NK) cells play an important role in the innate immune response. The summation of activation and inhibitory signals delivered through cell surface membrane receptors determines NK cell function. However, the continuous engagement of an activating receptor on NK cells appears to render the cells hyporesponsive to stimulation through other unrelated activating receptors. The mechanism by which this takes place remains unclear. Herein we demonstrate that continuous *in vivo* engagement of the Ly49H receptor with its ligand, m157, results in Ly49H^+^ NK cells that are hyporesponsive to further stimulation by other ITAM-dependent and independent receptors, while Ly49H^−^ NK cells remain unaffected. The hyporesponsiveness of the NK cell correlates with the degree of Ly49H receptor downmodulation on its cell surface. We observe defects in calcium flux in the hyporesponsive NK cells following stimulation through the NK1.1 receptor. In addition, we observe differences in signaling molecules that play a role in calcium flux, including spleen tyrosine kinase (Syk) at baseline and phosphorylated phospholipase C gamma 2 (p-PLCγ2) at both baseline and following stimulation through NK1.1. We also demonstrate that various ITAM associated activation receptors, including Ly49H, remain associated with their respective adaptor molecules. With regard to *in vivo* NK cell function, we did not find differences in the formation of metastatic lung lesions following IV injection of B16 melanoma cells. However, we did observe defects in rejection of missing-self targets *in vivo*. The data suggest that continuous engagement of the Ly49H activating receptor on NK cells results in hyporesponsiveness of the NK cells to all of the ITAM-dependent and independent receptors we analyzed due to altered signaling pathways downstream of the receptor and adaptor molecule.

## Introduction

NK cells effector function (release of cytokines and cytotoxic granules) is determined by the summation of stimulation received through activating and inhibitory receptors expressed on its cell surface (1–3). Several NK cell-activating receptors signal through immunoreceptor tyrosine-based activation motif (ITAM) bearing adaptor molecules. These include Ly49H and NK1.1, which associate with DAP12 and FcRε1γ, respectively. Engagement of the activating receptor results in phosphorylation of tyrosine residues within the ITAM motif, which serve as binding sites for Syk and/or Zap70, causing a downstream signaling cascade (4). Others and we have demonstrated that continuous engagement of the activating receptor results, counterintuitively, in the hyporesponsiveness of murine and human NK cells (5–8). Although this appears to be an important mechanism in determining NK responsiveness, we do not yet clearly understand the changes that take place within these cells following continuous engagement of an activating receptor.

Prior studies looking at lymphokine activated killer (LAK) cells suggest that continuous engagement of NKG2D with its ligand H60 *in vitro* results in dissociation from its adaptor molecule DAP12. In addition, the LAK cells demonstrated a decrease in the intracellular level of a number of ITAM-associated adaptor molecules, including DAP12 and CD3ζ (9). Continuous engagement of NKG2D on LAK cells *in vitro* also resulted in defective Ca^2+^ mobilization following stimulation through multiple activating receptors including NK1.1, NKp46, and CD16, but not Ly49D (9, 10). Furthermore, continuous engagement of NKG2D on LAK cells resulted in defective killing of H60 expressing and RMA/s (missing-self) targets, as well as attenuated antibody-dependent cellular cytotoxicity (ADCC), but no defect in killing of CHO (Ly49D ligand expressing target) cells. Finally, continuous *in vitro* engagement of Ly49D on LAK cells (by incubation with CHO cells) resulted in Ly49D^+^, but not Ly49D^−^, LAK cells having defects in killing H60 expressing targets, RMA/s targets, and CHO cell targets as well as antibody-dependent cell cytotoxicity (10). This suggests a selectivity in how continuous engagement of activating receptors results in defects to other activating receptors.

*In vivo* studies addressing the continuous engagement of NKG2D, using a transgenic mouse in which human MICA is ubiquitously expressed under the MHC class I promoter, conflict with prior *in vitro* findings (11). Defects in NK1.1- and Ly49D-mediated killing, but not NKp46-mediated killing, were seen following chronic NKG2D engagement. However, IFNγ production following stimulation with plate bound NK1.1 and NKp46, but not Ly49D, revealed increased production of the cytokine by NK cells where continuous engagement of the NKG2D receptor took place compared to WT NK cells (11). Thus, based on this prior study, the continuous engagement of NKG2D in this system appeared to have opposite effects on killing and IFNγ production mediated by other activating receptors.

Several studies have attempted to address the role continuous engagement of NKG2D plays in the NK cell response to melanoma tumors *in vivo*. DNAM1 and natural cytotoxicity receptors (NCRs) have been shown to be important for NK cell mediated immunity to melanoma cells, as ligands for NKP46, NKG2D, and DNAM1 appear to be expressed on these malignant cells (12–14). There was no difference in growth rate of melanoma tumors following subcutaneous injection of B16-F10 melanoma cells in MICA-expressing transgenic mice compared to litter mate control mice (11). Another study demonstrated that melanoma tumor cells engineered to secrete a soluble form of MULT1 were rejected better by NK cells than the parental melanoma cell line, suggesting the soluble MULT1 increases tumor rejection by enhancing NK cell function (15). They hypothesize that expression of the NKG2D ligand on non-tumor cells within the tumor environment results in desensitization of the NK cells due to continuous engagement and downregulation of the NKG2D receptor. The soluble MULT1 blocks these interactions, resulting in an increase of the NK cell responsiveness to the tumor. Thus, the precise role that continuous engagement of NKG2D *in vivo* plays in the NK cell response to melanoma challenge, as well as the particular activating receptors involved in this response (NKp46, DNAM1, or others), is not entirely clear.

In this manuscript, we aim to shed light on the mechanism by which continuous engagement of NK cell activating receptors results in NK cell hyporesponsiveness, as well as how this impacts NK cell response to tumor cells *in vivo*. To accomplish this, we employed a transgenic mouse model developed in our laboratory that ubiquitously expresses the murine cytomegalovirus protein m157 under the control of the β-actin promoter (7, 16). In this mouse (m157Tg), the continuous engagement of m157 with Ly49H results in systemic Ly49H downregulation and dysfunction. Using this model, we demonstrate NK cells undergoing continuous engagement of the Ly49H receptor are hyporesponsive to further stimulation by a wide range of ITAM-dependent and independent receptors. This hyporesponsiveness correlates with the degree of Ly49H downmodulation. Hyporesponsive NK cells demonstrated defective calcium flux upon stimulation through the NK1.1 activating receptor. In addition, we observed differences in the signaling molecules involved in mediating calcium flux, including Syk and p-PLCγ2. We did not observe any discrepancies in the intracellular levels of ITAM-containing adaptor molecules, and they appeared to remain associated with their respective activating receptors. *In vivo*, we detected defects in killing of missing-self targets but did not find differences in the formation of metastatic lung lesions following IV injection of B16 melanoma cells. The data suggest that the continuous engagement of this activating receptor on NK cells results in hyporesponsiveness of the NK cells to multiple ITAM-dependent and independent receptors due to changes in downstream signaling pathways.

## Materials and Methods

### Mice

The B6.ROSAm157 mouse was generated by cloning the Smith strain MCMV m157 DNA, obtained by PCR with NotI and XhoI ends, into pBigT to generate pBigTm157. This construct was digested with PacI and AscI and inserted into pROSA26-PA to generate pROSAm157. The plasmids pBigT and pROSA26-PA were gifts from Frank Costantini (Addgene plasmids # 21270 and # 21271; RRID:Addgene_21270 and RRID:Addgene_21271) (17). The plasmid pROSAm157 was linearized with BbvCI and used to target the Rosa locus in B6 Blu ES cells. Targeted clones were injected into albino B6 host blastocysts using standard methods. High coat color chimeric mice were bred to establish germline transmission. B6.ROSAm157 was bred to B6.C-Tg(CMV-cre)1Cgn/J or B6.Cg-Ndor1Tg(UBC-cre/ERT2)1Ejb/2J mice. The generation of a transgenic mouse constitutively expressing m157 (m157Tg) was previously described (7). During the generation of this line, a second founder that expressed lower levels of m157 was also obtained (m157Tg.Low). Mice were maintained under specific pathogen-free conditions and used after they reached 8 weeks of age. All animals received humane care according to the criteria outlined in the “Guide for the Care and Use of Laboratory Animals” prepared by the National Academy of Sciences and published by the National Institutes of Health (NIH publication 86-23 revised 1985). The Animal Studies Committee at Washington University (St. Louis, MO) approved all animal studies.

### Tamoxifen Treatment and NK Cell Depletion of Mice

Mice were administered tamoxifen from Toronto Research Company (Toronto, Canada) by intraperitoneal injection (2.5 mg/mouse/d for 3–5 consecutive days) in 100 μl of sunflower seed oil (Sigma-Aldrich) as according to published protocols (18). For *in vivo* killing experiments, NK cells were depleted from a subset of mice by intraperitoneal injection of 100 μg α-NK1.1 (pk136) antibody on days −5 and −2. For NK cell depletion prior to melanoma injection, mice were treated on days −4 and +3.

### Antibodies and Flow Cytometry

Except where otherwise indicated, all antibodies were obtained from Biolegend (San Diego, CA). Cell surface receptors were labeled with NK1.1 (pk136), Ly49D (4E4, a gift from Wayne Yokoyama), Ly49H (3D10), NKp46 (29A1.4), CD49b (DX5), DNAM1 (10E5), IL2 receptor (IL-2Rβ), and CD3 (17A2). For intracellular staining of Syk and Zap70, cell surface receptors were stained and the cells were fixed with BD Cytofix/Cytoperm, the cells were permeabilized with 1% saponin in flow cytometry buffer and stained intracellularly for Syk (5F5) and Zap70 (1E7.2). For intracellular staining of PLCγ2, cells were initially stained with α-Ly49H-AF647 (3D10) and α-CD49b-BV421 (DX5). The cells were fixed in 3% paraformaldehyde in PBS and then permeabilized with absolute methanol followed by staining with α-NKp46-PerCP-eFluor710 (29A1.4) from Thermo Fisher Scientific (Waltham, MA), α-CD3-PE-Cy7 (17A2), α-NK1.1-AF488 (pk136), and α-PLCγ2-PE obtained from Miltenyi (Bergisch Gladbach, Germany). Samples were run on a BD FacsCanto, BD LSR-II, or BD Fortessa X20 (Becton Dickinson, Franklin Lakes, NJ) and analyzed using FlowJo v10.5.3 (Becton Dickinson).

### Activation Assays

Twelve well tissue culture treated plates (TPP, Trasadingen, Switzerland) were incubated at 37°C for 90–120 min with antibody diluted in PBS. Dilutions were as follows: α-NK1.1 (pk136) at 8 μg/ml, α-NKp46 (29A1.4) at 4 μg/ml, α-Ly49D (4E5) at 32 μg/ml, and α-DNAM1 (10E5) at 500 ng/ml. Background levels of IFNγ and CD107a were measured using PBS only coated plates. Freshly isolated splenocytes were depleted of erythrocytes by ammonium chloride lysis, resuspended in dye-free RPMI-1640 with 10% FBS and 5 mM HEPES, and filtered through 110 um nylon mesh. After washing the wells, 5 × 10^6^ cells were loaded onto each well at 10^7^ cells/ml and incubated at 37°C in a 5% CO_2_ incubator. The α-NKp46 and α-DNAM1 plates (and their respective controls) were co-stimulated with 400 IU/ml of IL2 (Chiron, Emeryville, CA). Other samples were mono-stimulated with IL2 at 2,000 IU/ml or IL15 at 125 ng/ml (Peprotech, Rocky Hill, NJ). After 1 h, 200 ng/ml α-CD107a-APC (1D4B), 5 μg/ml Brefeldin A, and 2 μM monensin (all from Biolegend, San Diego, CA) were added to label surface-exposed Lamp1 and prevent excretion of IFNγ. The assay was further incubated for 7 h prior to being stopped by the addition of cold flow cytometry buffer (PBS with 1% FBS and 5 mM NaN_3_). Fc receptors were blocked with supernatant from the hybridoma 2.4G2, and cells were surface stained with biotinylated α-Ly49H, streptavidin-PE, α-CD49b-BV421, α-NKp46-PerCP-e710, and α-CD3-PE-Cy7. For stimulation through Ly49D, DNAM1, IL2 receptor and IL15 receptor, NK cells were identified as NK1.1^+^, NKp46^+^, CD3^−^. For stimulation through NKp46, NK cells were identified as NK1.1^+^, CD49b^+^, CD3^−^. For stimulation through NK1.1, NK cells were identified as CD49b^+^, NKp46^+^, CD3^−^. Following fixation with BD Cytofix/Cytoperm, the cells were permeabilized with 1% saponin in flow cytometry buffer and stained intracellularly with α-IFNγ-AF488 (XMG1.2). The samples were analyzed on a BD FacsCanto flow cytometer (Becton Dickinson, Franklin Lakes, NJ).

### Ca^2+^ Flux

Freshly-isolated splenocytes (5 × 10^6^ cells/ml) were dye-loaded for 30 min at 37°C in serum-free, phenol red-free RPMI with 0.002% Plurionic F127 and the calcium-sensitive dye, Indo1 AM (Invitrogen, Eugene OR), at 250 nM from a 2.5 mM stock in DMSO. After washing, the cells were stained on ice for 30 min at 5 × 10^7^ cells/ml with α-Ly49H-AF647 (clone 3D10), α-CD49b-PE (DX5), α-NK1.1-PerCP-Cy5.5 (pk136), and α-CD3-PE-Cy7 (17A2). The cells were then washed and stored on ice in the dark. Subsequently, 500 μl aliquots of cells (5 × 10^6^/ml) were warmed to 37°C for exactly 5 min in assay buffer (dye-free RPMI 1640 with 10% FBS and 5 mM HEPES). To establish the baseline ratio of filters 379/28 and 525/50, the samples were analyzed for 45 s on either a BD LSR-II or a BD Fortessa X20 on low speed, each equipped with a custom-made sample heater set to 37°C. To this was added 8 μl of either assay buffer alone or antibody [α-NK1.1-biotin (pk136), α-Ly49H-biotin (3D10), or α-Ly49D-biotin (4E4)] at 500 μg/ml for 45 s, followed by 32 μl of 4 mg/ml streptavidin to crosslink the receptors. After 3 min, 5 μl of 200 μg/ml ionomycin was added to each sample to verify the maximum potential Ca^2+^ release. The percentage of NK cells with Ca^2+^ signal above the initial baseline was calculated for a 45 s window following crosslinking, and the ratio of this percentage in Ly49H^+^ to Ly49H^−^ cells was plotted. The peak/basal intensity and area under the curve were calculated for a 100 second window using FlowJo.

### Immunoprecipitation and Western Blotting of Activating Receptors

Pools of NK cells from 10 to 20 mice were enriched by negative selection using either Mojosort (Biolegend, San Diego, CA, USA) or EasySep (Stem Cell Technologies, Vancouver, Canada) mouse NK cell isolation kits. For some experiments, cells were sorted at 4°C for CD49b-BV421^+^, NKp46-PerCP-e710^+^, CD3-AF488^−^, Ly49H-AF647^+^/Ly49H-AF647^−^. Cells were then lysed in maltoside buffer (1% n-Dodecyl beta-D-maltoside, 100 mM HEPES, 150 mM NaCl, 10% glycerol, pH 7.4) with Thermo Halt protease and phosphatase inhibitors (Thermo Fisher Scientific, Waltham, MA) for 1 h at 4°C and 3.3 × 10^6^ cells/ml prior to spinning at 20,000 × *g* for 10 min. Pierce protein A + G beads (Thermo Fisher Scientific, Waltham, MA) were pre-saturated with 50 μg of antibody per 500 μg of beads for 1 h at RT. After washing the beads, the cell lysate supernatant was incubated with the magnetic beads for 1 or 2 h at 4°C with agitation. The beads were then washed five times in phosphate buffered saline with 0.05% Tween-20 while the lysate was transferred to the next set of antibody-labeled bead in the order α-Ly49H → α-Ly49D → α-NK1.1. The precipitated complexes were eluted from the beads using Thermo Bolt sample loading dye at 37°C for 20 min, then boiled, separated by SDS-PAGE, blotted onto nitrocellulose, and probed with α-Dap12 rabbit polyclonal antibody gifted by T. Takai (19, 20) or α-FcεR1γ (MilliporeSigma, Burlington, MA, USA).

Total cell lysates were prepared and sorted as above, separated by western blot, and probed with α-βActin (Santa Cruz Biotechnology, Dallas, TX), α-CD3ζ (6B10.2), α-Dap12 rabbit polyclonal antibody, or α-FcεR1γ.

### Analysis of PhosphoPLCγ2

Splenocytes were stained on ice for 30 min with α-Ly49H-AF647 (clone 3D10) and α-CD49b-BV421 (clone DX5). After washing off excess antibody, 100 μl aliquots of cells (2 × 10^7^/ml) were warmed to 37°C for exactly 5 min in assay buffer (dye-free RPMI 1640 with 10% FBS and 5 mM HEPES). To this was added 50 μl of either assay buffer alone or α-NK1.1-biotin (clone pk136) at 96 μg/ml for 45 s, followed by 50 μl of 128 μg/ml streptavidin (Leinco, St. Louis, MO) for 45 s. The reaction was quenched by the addition of 1 ml of 3% paraformaldehyde in PBS for 15 min. The cells were then permeabilized with absolute methanol for 30 min on ice and stained for 1 h with α-NKp46-PerCP-eFluor710 (29A1.4), α-CD3-PE-Cy7 (17A2), α-NK1.1-PE (pk136), and α-phosphoPLCγ2-AF488 (Y759, clone K86-689.37). The α-phosphoPLCγ2 was purchased from Becton Dickenson (Franklin Lakes, NJ). For experiments where α-PLCγ2 was used, the panel was modified to also include α-PLCγ2 (Miltenyi, Bergisch Gladbach, Germany), αNK1.1-PE-Cy7 (pk136), and α-CD3-BV510 (17A2). Samples were analyzed on a BD Fortessa X-20 and gated for NK cells. The GMFI of the phosphoPLCγ2 signal from the mock-stimulated cells was subtracted from those treated by crosslinking NK1.1, and the ratios of the phosphoPLCγ2 signals from Ly49H^+^ vs. Ly49H^−^ cells from each mouse were plotted.

### Tumor Inoculation

B16-F10 melanoma cells were grown at 37°C in a 5% CO_2_ incubator in DMEM media supplemented with 10% fetal bovine serum to <70% confluency, removed with minimal exposure to trypsin and EDTA, washed three times in PBS, and resuspended in DMEM with 10 mM HEPES. After filtering through a 40 μm mesh filter, the cells were kept on ice until use. Varying doses of melanoma cells were injected into the lateral tail vein in 200 μl volumes. After 14 days, the lungs were harvested and fixed in Fekete's solution overnight. Following exchange into PBS, the lungs were dissected into five lobes and all visible melanoma lesions were enumerated under magnification.

The B16-F1.1 cell line was generated by passing the poorly colonizing line B16-F1 into a WT mouse. At 14 days, the melanoma engraftments were harvested and mechanically homogenized using a scalpel to mince and a 40 μm mesh filter to remove large pieces of tissue. This culture was expanded *in vitro* until it became morphologically homogenous.

### *In vivo* Killing Assays

Donor splenocytes obtained from WT, m157Tg and H2Kb^−/−^ Db^−/−^ (KODO) (21) mice were harvested and labeled *in vitro* with different combinations of CFSE (Life Technologies), CellTrace Violet, and CellTrace Far Red (Thermo Fisher). Recipient mice were injected IV with 1 × 10^6^ of each donor cell. Spleens from recipient mice were harvested 24 h after transfer of donor cells. NK cell-specific rejection was calculated by gating on CD19+ transferred cells. Rejection was quantified as %Rejection = [1–(Target/Control)/(Target/Control)_Average(NKdepleted)_]×100, where the target was the donor cell of interest, and the control was a WT donor cell population. The ratio of target to control cells was normalized to the average ratio recovered from NK cell-depleted mice to calculate rejection by NK cells.

### Statistics

Statistical analyses as detailed in figure legends were performed using Prism 6 (GraphPad, San Diego, CA, USA) and Excel (Microsoft, Redmond, WA, USA). The *p*-values for all figures, except for Figure 2, were calculated using the Student's *t*-test with Welch's correction. For Figure 2, statistical analysis was performed using a Pearson correlation test.

## Results

### Continuous Engagement of the Ly49H Receptor Results in NK Cell Functional Defects Following Stimulation Through Both ITAM-Dependent and Independent Activating Receptors

**Figure 1:**
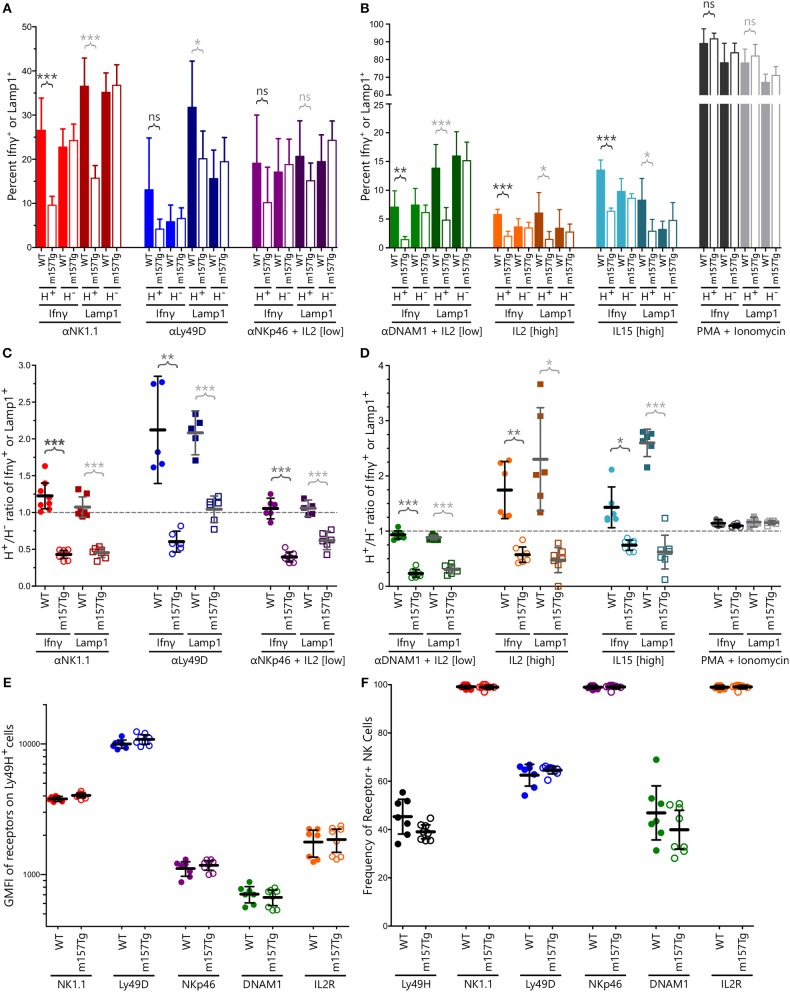
Continuous engagement of Ly49H results in NK cell functional defects following stimulation through multiple ITAM-dependent and independent activating receptors without altering their cell surface expression levels. **(A,B)** The percentage of Ly49H^+^ and Ly49H^−^ NK cells expressing IFNγ or Lamp1 above background following stimulation of WT and m157Tg splenocytes through **(A)** the ITAM-dependent receptors NK1.1, Ly49D, and NKp46 or **(B)** the ITAM-independent receptors DNAM1, IL2, and IL15 as well as with PMA and ionomycin. **(C,D)** The Ly49H^+^/Ly49H^−^ ratio of IFNγ or Lamp1 producing NK cells using values generated from **(A)** and **(B)**. **(E)** GMFI of indicated activating receptors on Ly49H^+^ NK cells from m157Tg and WT mice. **(F)** Frequency of various receptor positive NK cells from WT and m157Tg mice. Ly49H is gated on total NK cells while all of the other receptors are gated on Ly49H^+^ NK cells. All results are presented as the mean ± 95% CI (*p < 0.05; **p < 0.005; ***p < 0.0005; ns, not significant). For panels **(A–F)**, *n* ≥ 5, each combined from at least two independent experiments. Each data point in panels **(C–F)** represents an individual mouse.

To investigate how continuous engagement of Ly49H alters NK cell function, we measured intracellular levels of IFNγ and surface-exposed Lamp1 (a surrogate marker for degranulation) following *in vitro* stimulation of WT and m157Tg splenocytes through multiple different receptors. Representative plots of stimulation through NK1.1 are shown in Supplemental Figure 1. A significantly smaller percentage of Ly49H^+^ NK cells from m157Tg mice expressed Lamp1 compared to WT Ly49H^+^ NK cells when stimulated through NK1.1 or Ly49D alone but not NKp46 in the presence of low levels of IL2 (Figure 1A). A significantly smaller percentage of Ly49H^+^ NK cells from m157Tg mice expressed IFNγ following stimulation through only NK1.1 (Figure 1A). As expected, there was no difference when we compared IFNγ or Lamp1 expression in Ly49H^−^ NK cells from both mice (Figure 1A). We observed the Ly49H^+^/Ly49H^−^ NK cell ratio for IFNγ and Lamp1 expression to be lower in m157Tg mice compared to WT mice when the splenocytes were stimulated though any of the three receptors, as this ratio controls for variation in NK cell activation potential between different mice (Figure 1C). Thus, continuous engagement of the Ly49H receptor results in IFNγ production and degranulation defects upon stimulation of these NK cells through other ITAM-dependent activating receptors.

We also assessed NK cell function in response to stimulation through ITAM-independent receptors including DNAM1 (in the presence of low levels of IL2), IL2 receptor, IL15 receptor, and the combination of phorbol 12-myristate 13-acetate (PMA) and ionomycin (Figures 1B,D). Similar to ITAM-dependent receptors, we observed a significantly smaller percentage of Ly49H^+^ NK cells expressing IFNγ or Lamp1 from m157Tg compared to WT mice following stimulation *in vitro* (Figure 1B). Again, there was no difference when we compared IFNγ or Lamp1 expression in Ly49H^−^ NK cells from both mice (Figure 1B). Stimulation through any of these three ITAM-independent receptors resulted in a significantly lower Ly49H^+^/Ly49H^−^ NK cell ratio in terms of IFNγ and Lamp1 expression when comparing m157Tg and WT mice (Figure 1D). We observed no difference when the cells were stimulated with PMA and ionomycin, suggesting that receptor engagement is important for the phenotype (Figures 1B,D).

To determine if functional differences were simply due to alterations in activating receptor abundance on the cell surface, we assessed these levels on unstimulated NK cells by flow cytometry (Figure 1E). We also assessed the percentage of Ly49H^+^ NK cells, as well as the percentage of Ly49H^+^ NK cells expressing the various receptors, from WT and m157Tg mice (Figure 1F). We did not observe a difference in the geometric mean fluorescence intensity (GMFI) of these activating receptors when we compared expression levels on Ly49H^+^ NK cells from m157Tg and WT mice, making it unlikely that alteration in receptor levels explains the functional differences we observed. In addition, we did not observe a significant difference in the percentage of NK cells expressing any of the receptors, including Ly49H, when we compared WT and m157Tg mice. This suggests that the number of Ly49H^+^ NK cells does not change in the m157Tg mice.

### NK Cell Hyporesponsiveness Correlates With Downmodulation of Ly49H

**Figure 2:**
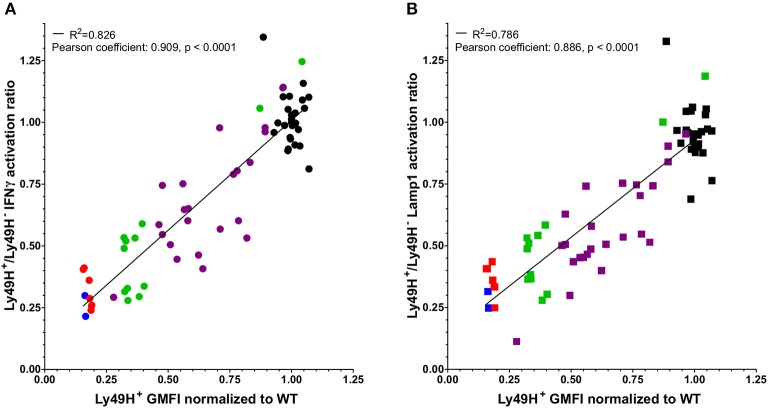
Defects in NK cell function correlate with downmodulation of the Ly49H receptor. The Ly49H^+^/Ly49H^−^ ratio of IFNγ **(A)** or Lamp1 **(B)** producing NK cells from 26 WT mice (black) and 45 mice that ubiquitously express m157 [7 m157Tg (red), 2 m157Tg.Low (blue), 24 UBC-Cre-ERT2 × ROSAm157Flox (purple), and 12 hCMV-Cre x ROSAm157Flox (green)] following stimulation through NK1.1 plotted against the GMFI of the Ly49H receptor normalized to WT GMFI of Ly49H on NK cells for a given experiment. Data were collected across nine independent experiments. Each data point represents an individual mouse. The best fit lines were generated by linear regression using Prism 6. The *R*^2^ value is shown in each panel. Correlation of the two variables was determined using a Pearson test. The Pearson coefficients and *p*-values are shown in each panel.

Engagement of the Ly49H receptor with m157 results in the downmodulation of the receptor (7, 16, 22). In addition, we noticed differences in Ly49H cell surface expression among various lines of m157-expressing mice. To determine if the extent of downmodulation of the receptor correlates with decreased NK cell function, we compared the normalized GMFI of Ly49H to the Ly49H^+^/Ly49H^−^ NK cell ratio of IFNγ production and Lamp1 expression in response to NK1.1 engagement in various mice that ubiquitously expressed m157 (Figures 2A,B). Due to staining and instrument variation, we normalized Ly49H GMFI values to the average of WT cells in a given set to allow comparisons between experiments performed at different times. Our results demonstrate that decreased NK cell function was associated with the expression of lower levels of the Ly49H receptor and there was a strong positive correlation between Ly49H expression levels and Ly49H^+^ NK cell function. We hypothesize that more extensive or frequent binding of the ligand (m157) with the receptor (Ly49H) results in lower levels of Ly49H expression on the NK cell and its subsequent decreased function.

### NK Cells From m157Tg Mice Exhibit Altered Calcium Flux Following Cross-Linking of NK1.1

Altered degranulation and IFNγ production in Ly49H^+^ NK cells from m157Tg mice following stimulation through various activating receptors suggests a defect in intracellular signaling within these NK cells. Furthermore, the administration of PMA and ionomycin (which results in activation of protein kinase C and the rapid increase of cytoplasmic Ca^2+^ concentrations, respectively) did not alter IFNγ production or Lamp1 expression in Ly49H^+^ NK cells from WT mice compared to m157Tg mice. Since PMA and ionomycin function downstream of receptor engagement and bypasses normal calcium flux, it suggests that there should be a defect in calcium flux following receptor engagement in the hyporesponsive NK cells.

**Figure 3:**
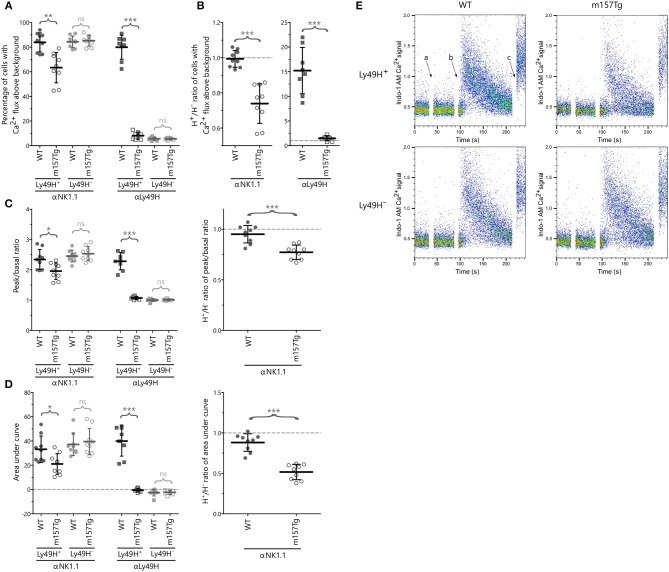
NK cells from m157Tg mice exhibit altered calcium flux following cross-linking of NK1.1. **(A)** Percentage of Ly49H^+^ and Ly49H^−^ NK cells from WT and m157Tg mice with calcium flux above background following crosslinking of the NK1.1 and Ly49H receptors. **(B)** The Ly49H^+^/Ly49H^−^ ratios of the data from panel **(A)**. **(C)** The ratio of the peak to basal mean flux and **(D)** the area under the curve (AUC) following stimulation through NK1.1 and Ly49H in Ly49H^+^ and Ly49H^−^ NK cells. **(E)** Sample plots of Indo-1 AM Ca^2+^ signal vs. time in Ly49H^+^ and Ly49H^−^ NK cells from WT and m157Tg mice following NK1.1 crosslinking: (a) addition of b-pk136, (b) addition of streptavidin, and (c) addition of ionomycin. The results are presented as the mean ± 95% CI (*p < 0.05; **p < 0.005; ***p < 0.0005; ns, not significant). Data were collected across four independent experiments. Each data point represents an individual mouse.

To further evaluate these defects, we examined calcium flux (quantifying the percentage of stimulated NK cells with increased Indo-1 AM fluorescence blue shifting above resting background levels) within the Ly49H^+^ and Ly49H^−^ NK cells from WT and m157Tg mice following cross-linking of NK1.1 and Ly49H (Figures 3A,E). As expected, following crosslinking of NK1.1, we observed similar levels of calcium flux in Ly49H^−^ NK cells from both lines (Figure 3A). However, with the same stimulation, the Ly49H^+^ NK cell from m157Tg mice demonstrated decreased calcium flux compared to Ly49H^+^ NK cells from WT mice. Assessment of the Ly49H^+^/Ly49H^−^ NK cell ratio of calcium flux following the cross-linking of NK1.1, similar to our assessment of IFNγ and Lamp1, made the effect more pronounced by controlling for variation in NK cell activation potential between different mice (Figure 3B). This ratio is significantly lower in m157Tg mice, suggesting that there is a defect in calcium flux specific to Ly49H^+^ NK cells from m157Tg mice.

We also measured calcium flux in Ly49H^+^ and Ly49H^−^ NK cells from WT and m157Tg mice following cross-linking of Ly49H itself (Figures 3A,B). We would expect to see significantly higher calcium flux in Ly49H^+^ NK cells from WT compared to m157Tg mice due to the downmodulation of the Ly49H receptor seen in Ly49H^+^ NK cells from m157Tg mice. In addition, the presence of m157 binding to Ly49H in the m157Tg mice might prevent cross-linking of the receptor. The absence of Ly49H on Ly49H^−^ NK cells from WT or m157Tg mice would prevent any calcium flux in these NK cells. As expected, we only observed calcium flux above background in the Ly49H^+^ NK cells from the WT mice (Figure 3A).

To assess the intensity and duration of the calcium flux, we analyzed the ratio of the peak to basal mean flux and the area under the curve (AUC) following stimulation in Ly49H^+^ and Ly49H^−^ NK cells (Figures 3C,D). Although the peak to basal ratio and AUC appear lower in the Ly49H^+^ NK cells from m157Tg mice (Figures 3C,D), this is most likely due to the fact that fewer Ly49H^+^ NK cells flux in the m157Tg mice, as shown in Figure 3A, rather than a difference in intensity or duration. The cells that do not flux bring down the average calcium signal lowering the intensity peak and AUC. However, the Ly49H^+^ NK cells that flux in the m157Tg mice appear to do so at a similar intensity to Ly49H^+^ NK cells in the WT mice (Figure 3E).

### Continuously Stimulated NK Cells Display Altered Levels of Signaling Molecules Involved in Ca^2+^ Flux

Since Ly49H^+^ NK cells from m157Tg mice displayed defects in Ca^2+^ flux upon stimulation through NK1.1, we assessed ITAM-associated signaling proteins involved in Ca^2+^ mobilization. Phospholipase C-gamma (PLCγ), which exists in two isoforms, is an important regulator of Ca^2+^ mobilization in a number of immune cells. In NK cells, PLCγ2 appears to be the primary isoform involved in signal transduction following activation through engagement of certain activating receptors (23, 24). Studies by multiple groups have demonstrated that depletion of PLCγ2 resulted in impairment of NK cell function including cytotoxicity, IFNγ production, and calcium mobilization (23, 24). Thus, alterations in PLCγ2 and/or phosphorylated PLCγ2 (p-PLCγ2) levels in these NK cells could explain the altered Ca^2+^ flux and functional defects in the hyporesponsive NK cells.

**Figure 4:**
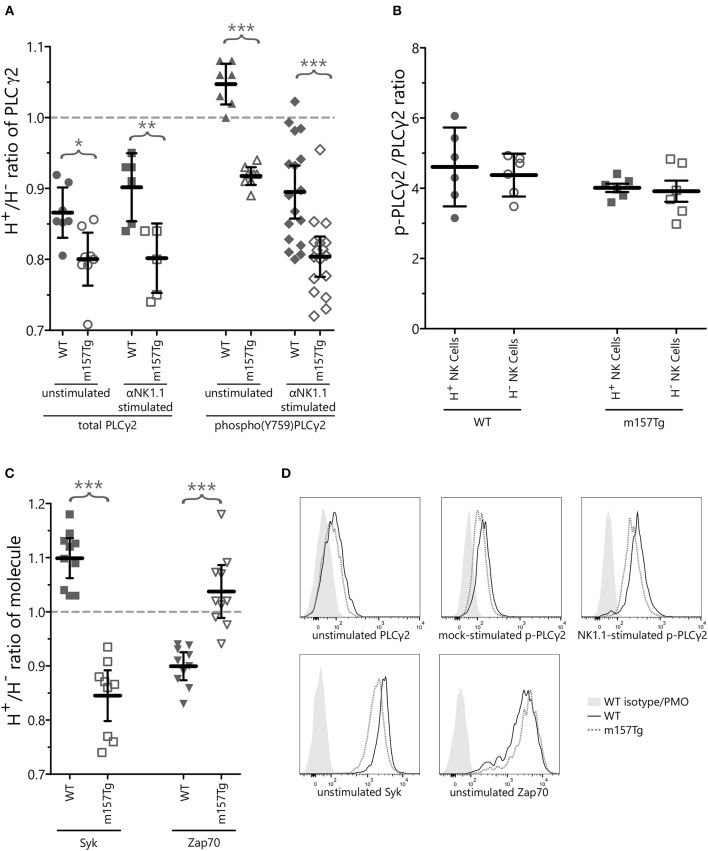
Continuously stimulated NK cells display altered levels of signaling molecules involved in Ca^2+^ flux. **(A)** The Ly49H^+^/Ly49H^−^ ratio of NK cells that are positive for intracellular PLCγ2 or phospho(Y759)PLCγ2 in WT and m157Tg mice at steady state or following crosslinking of the NK1.1 receptor. **(B)** The p-PLCγ2/ PLCγ2 ratio in Ly49H^+^ and Ly49H^−^ NK cells from WT and m157Tg mice following stimulation through NK1.1. **(C)** The Ly49H^+^/Ly49H^−^ ratio of NK cells that are positive for the intracellular expression of Syk or Zap70 in WT and m157Tg mice at steady state. **(D)** Sample histograms of intracellular levels of indicated proteins. Unstimulated, mock-stimulated (SA-PE only), and NK1.1-stimulated (b-pk136 → SA-PE) Ly49H^+^ NK cells from WT and m157Tg mice were used as indicated. Each histogram shows isotype staining or panel minus one (PMO), WT, and m157Tg. The results are presented as the mean ± 95% CI (*p < 0.05; **p < 0.005; ***p < 0.0005). The data were collected from at least two independent experiments. Each data point represents an individual mouse.

We observed a small but statistically significant reduction of the Ly49H^+^/Ly49H^−^ ratio of the GMFIs of total PLCγ2 as well as p-PLCγ2 in unstimulated NK cells from m157Tg compared to WT mice (Figures 4A,D). In addition, after subtracting the steady-state background from mock stimulated cells, we observed a further small but significant decrease in the Ly49H^+^/Ly49H^−^ ratio of p-PLCγ2 in NK cells from m157Tg compared to WT mice following cross-linking of the NK1.1 receptor (Figures 4A,D). Finally we assessed the p-PLCγ2/ PLCγ2 ratio and observed that the ratio was the same in both subsets of NK cells from WT and m157Tg mice following stimulation through NK1.1 (Figure 4B). Taken together, this suggests that reduction in total PLCγ2 is predominantly responsible for the lower p-PLCγ2 levels seen in Ly49H^+^ NK cells from m157Tg mice.

Zeta-chain-associated protein kinase 70 (Zap70) and Syk are two kinases, downstream of ITAM-containing adaptor molecules, involved in initiating the intracellular phosphorylation cascade following engagement of certain activating receptors on the surface of the NK cells. A prior study identified a subset of human NK cells that expressed low levels of the Zap70 and Syk kinases (Zap70^low^Syk^low^) and had decreased functional potential compared to Zap70^hi^Syk^hi^ NK cells. In addition, they showed that extended NK cell activation resulted in the loss of Zap70 and Syk (25). This suggests that extended NK cell activation leads to low levels of Syk and Zap70, resulting in decreased NK cell function.

To determine if loss of Zap70 or Syk plays a role in the decreased function of Ly49H^+^ NK cells from m157Tg mice, we measured intracellular level of these kinases within Ly49H^+^ and Ly49H^−^ NK cells from WT and m157Tg mice (Figures 4C,D, Supplemental Figure 2). When we assessed the Ly49H^+^/Ly49H^−^ NK cell ratio of intracellular Syk levels, there was a small but statistically significant decrease in m157Tg compared to WT mice (Figure 4C). Similar to the human NK cell subset, Syk levels appear to be lower in the Ly49H^+^ NK cells from m157Tg mice, presumably because of continuous engagement of Ly49H with m157. As opposed to the human NK cells, we did not observe a decrease of Zap70 levels in m157Tg mice compared to WT mice. In fact, there was a very small but statistically significant increase in Zap70 in Ly49H^+^ NK cells from m157Tg mice (Figure 4C). Thus, continuous engagement of Ly49H with m157 appears to decrease Syk but not Zap70 in these NK cells.

### Intracellular Levels of Adaptor Molecules Within NK Cells Are Unchanged and Activating Receptors Remain Associated With Their Adaptor Molecules Following Continuous Engagement of the Ly49H Receptor

**Figure 5:**
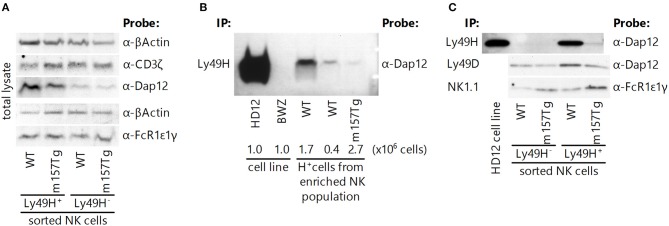
Intracellular levels of adaptor molecules within NK cells are unchanged, and activating receptors remain associated with their adaptor molecules following continuous engagement of the Ly49H receptor. **(A)** Western blot analysis of total adaptor protein levels (CD3ζ, DAP12, and FcRε1γ) in Ly49H^+^ and Ly49H^−^ NK cell lysate from WT and m157Tg mice. **(B)** Western blot of DAP12 following immunoprecipitation of Ly49H from cell lysates made from the HD12 cell line (BWZ cells that overexpress Ly49H and DAP12) and the parental BWZ cell line, as well as NK-enriched splenic cells from WT and m157Tg mice. The numbers below the enriched NK cell lanes indicate the quantity of Ly49H^+^ NK cells loaded. **(C)** Western blot of the indicated adaptor molecule following immunoprecipitation with its associated receptor from Ly49H^+^ and Ly49H^−^ splenic NK cell lysate from WT and m157Tg mice. For each of the blots, one representative of at least two experiments is shown.

A previous study suggests that intracellular levels of some ITAM-bearing adaptor molecules decrease and that they may dissociate from their receptors following continuous engagement of an activating receptor on the NK cell (9). Phosphorylation of the ITAM motif within these adaptor molecules appears to create binding sites for Syk and Zap70 (26–28). It is possible that Syk, bound to ITAM-bearing adaptor molecules, could undergo the same process that causes a decrease in levels of the intracellular adaptor molecules. To determine if decreased intracellular levels of Syk were a result of its loss due to association with ITAM-bearing adaptor molecules, we assessed the levels of ITAM-bearing adaptor molecules in Ly49H^+^ and Ly49H^−^ NK cell lysates from WT and m157Tg mice. We observed similar levels of the adaptor molecules DAP12, CD3ζ, and FcRε1γ in either Ly49H^+^ or Ly49H^−^ NK cells when we compared WT and m157Tg mice (Figure 5A). Therefore, decreases in total levels of these particular adaptor molecules within the NK cell is unlikely to explain the hyporesponsiveness of Ly49H^+^ NK cells from m157Tg mice.

To ascertain if dissociation of the receptors with their cognate adaptor molecules was responsible for the hyporesponsiveness of the Ly49H^+^ NK cells in m157Tg mice, we examined these associations by immunoprecipitation. We observed that NK1.1 and Ly49D remained associated with FcRε1γ and DAP12, respectively, in Ly49H^+^ NK cells from both WT and m157Tg mice (Figure 5C). Furthermore, we were able to demonstrate that the Ly49H receptor remained associated with DAP12 in Ly49H^+^ NK cells from m157Tg mice when we used substantial numbers of Ly49H^+^ NK cells from the m157Tg mice (Figures 5B,C). Presumably, the requirement for more cells to detect DAP12 bound to Ly49H is due to the lower apparent expression of Ly49H on these NK cells as assessed by flow cytometry. Alternatively, it is possible that a low frequency of Ly49H^+^ NK cells remain functional, and in these cells Ly49H remains associated with DAP12. While we can state that the various receptors remain associated with their respective adaptor molecules, we cannot quantitatively compare the amounts of adaptor associated with their receptors between the cell groups because western blot-compatible antibodies are not available to measure loading controls. Taken together, dissociation of adaptor molecules from their respective activating receptors fails to adequately explain the hyporesponsiveness of Ly49H^+^ NK cells from m157Tg mice.

### B16 Melanoma Clearance Is Unchanged in m157Tg Mice

**Figure 6:**
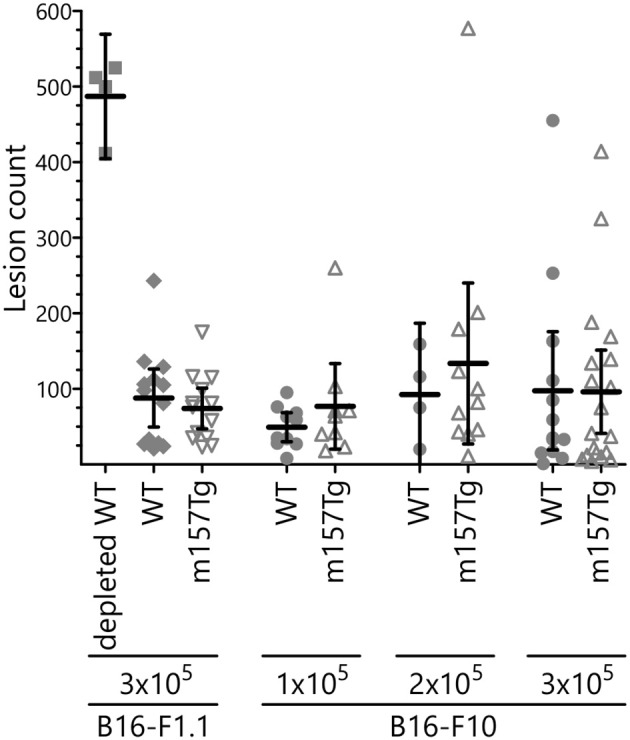
B16 melanoma clearance is unchanged in m157Tg mice. WT, m157Tg, and NK cell-depleted WT mice were intravenously injected with 3 × 10^5^ B16-F1.1 melanoma cells or 1 × 10^5^ to 3 × 10^5^ B16-F10 melanoma cells. Mice were euthanized on day 14 and tumor nodules on the outer surfaces of the lungs were counted. The results are presented as the mean ± 95% CI. There was no significant difference between WT and m157Tg recipient mice under any of the conditions. The data from B16-F1.1 and B16-F10 injections are combined from two and six independent experiments, respectively. Each data point represents an individual mouse.

NK cells play a key role in controlling tumor growth in the lung following tail vein injection of B16 melanoma cells into C57Bl/6 (B6) mice (12, 29). This is an NK cell dependent model in which depletion of NK cells results in dramatically increased lung and liver lesions. Upon injection into mice from our colony, the B16-F1 melanoma line, a well-established model used by other groups (29), failed to reliably produce pulmonary lesions (data not shown). We reasoned that, over the course of time, drift within our colony resulted in C57Bl/6 mice that were less permissive for growth of the B16-F1 melanoma cells. To address this, we isolated the rare lesions from a WT C57Bl/6 in our colony following injection with the B16-F1 melanoma cell line. This newly selected line was designated B16-F1.1. *In vivo* killing of this line is also NK cell dependent, as depletion of NK cells prior to IV injection resulted in a significantly larger number of lung lesions (Figure 6, Supplemental Figure 2). Following IV injection of the B16-F1.1 melanoma cells, we observed no significant difference in the number of lung lesions in m157Tg mice compared to WT B6 mice (Figure 6, Supplemental Figure 2). We also tested the widely available, more metastatic cell line B16-F10 (30). As with the B16-F1.1 melanoma line, B16-F10 did not exhibit any difference in lung lesion formation in m157Tg vs. WT mice following IV injection of various numbers of the tumor cells (Figure 6). This suggests that continuous m157/Ly49H engagement does not meaningfully alter NK cell response to melanoma cells *in vivo*.

### NK Cells From m157Tg Mice Are Less Efficient at Killing MHC Class I-deficient Target Cells *in vivo*

**Figure 7:**
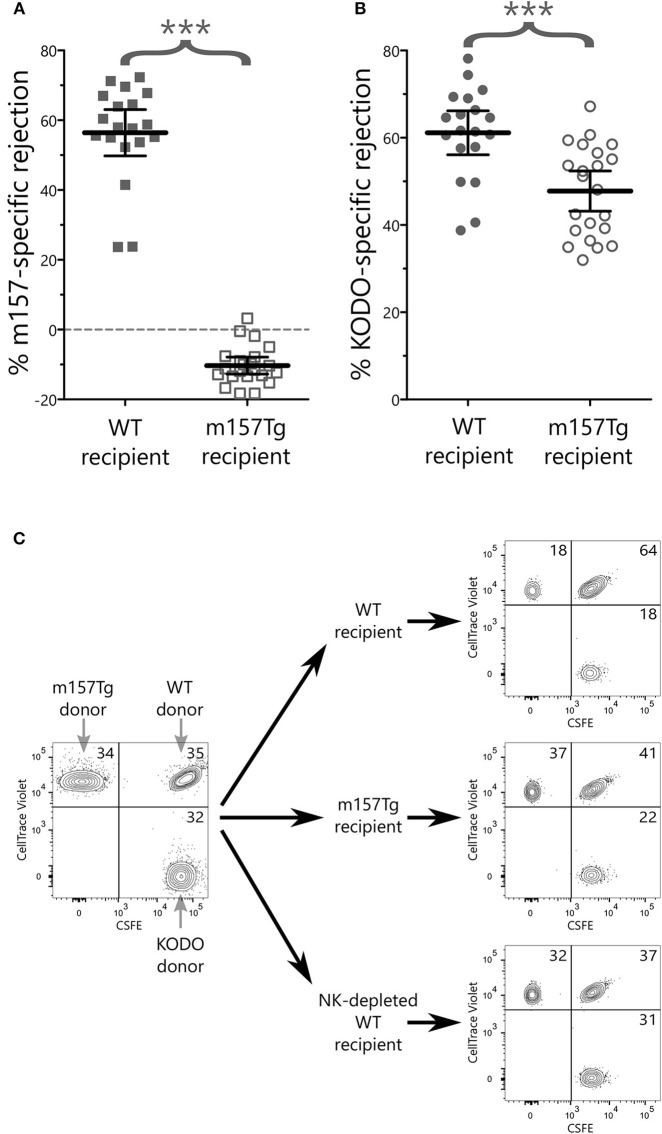
NK cells from m157Tg mice are less efficient at killing MHC class I-deficient target cells *in vivo*. Donor splenocytes from three target lines were differentially labeled with fluorescent dyes and injected into recipient mice with and without NK cell depletion. Twenty-four hours post injection, recipient spleens were analyzed for the presence or absence of donor target cells. **(A)** Percentage of NK cell-specific rejection of m157-expressiong donor splenocytes by WT vs. m157Tg recipient mice. **(B)** Percentage of NK cell-specific rejection of MHC class I-deficient donor splenocytes (KODO) by WT vs. m157Tg recipient mice. **(C)** Sample plots of donor cells used for *in vivo* killing assay. Specific rejection percentages were determined using the formula: 100 × [1–(Target/WT)/(Target/WT)_Average(NKdepletedWT)_]. The results are presented as the mean ± 95% CI (***p < 0.0005), and are combined from three independent experiments. Each data point represents an individual mouse.

To determine whether Ly49H^+^ NK cells from m157Tg mice exhibited any measurable *in vivo* defects, we performed *in vivo* cytotoxicity experiments to compare the function of NK cells from WT and m157Tg mice (31, 32). These experiments compared the killing of m157 expressing targets (splenocytes from m157Tg mice) or MHC class I-deficient targets (splenocytes from K^b^ × D^b^ deficient mice) to WT targets (splenocytes from WT mice) following injection into WT or m157Tg recipient mice. As expected, WT mice, but not m157Tg mice, effectively rejected m157Tg targets (Figures 7A,C). Additionally, m157Tg mice were less proficient at killing MHC class I-deficient targets when compared to WT mice (Figures 7B,C). This suggests that continuous engagement of m157/Ly49H generates defective NK cell responses to missing-self *in vivo*.

## Discussion

From the data herein, combined with previously published data, it is clear that continuous engagement of an activating receptor on the surface of NK cells results in downmodulation of the receptor as well as decrease in function of the NK cell in response to other stimuli (5–8). However, the changes taking place within the NK cell that result in its hyporesponsiveness remain unclear. Prior studies suggest a dissociation of the activating receptor with its adaptor molecule is responsible for the NK cell defects (9, 10). Another study suggests that continuous engagement of activating receptor might result in the differential expression of other activating receptors on the cell surface (11).

Through the assessment of Ly49H^+^ NK cells from m157Tg mice, where the continuous engagement of the Ly49H activating receptor takes place, we demonstrate that NK cell functional defects occur following stimulation through other ITAM-dependent and independent activating receptors. These include deficiencies in IFNγ production as well as degranulation (Figure 1). Of note, since NKp46 and DNAM1 stimulation took place in the presence of low levels of IL2, the observed effect is due to a combination of the two stimuli. In addition, the experiments described in this manuscript use total splenocytes in the stimulation assays, so defects seen following stimulation may not be NK cell intrinsic. Furthermore, we demonstrate that functional deficit correlates with the extent of Ly49H receptor downmodulation (Figure 2). Presumably, the variations in Ly49H levels observed are due to differences in the level of m157 expressed by the various ubiquitous promoters used to express m157, as well as variation in the degree of induction in the tamoxifen-inducible mice. This correlation suggests that engagement of the receptor with the ligand is important for eliciting the functional defect and that the frequency of the engagement influences NK cell function.

These results support the idea of an educational “rheostat” model of NK cell responsiveness in which the level of NK cell function is determined by the quantity of inhibiting and activating stimuli to which it is exposed during development (33–36). For example, a prior study demonstrated an inverse correlation between the levels of inhibitory receptor Ly49A expressed on the cell surface and the frequency of IFNγ production by Ly49A-monopositive cells stimulated through NK1.1, suggesting that the extent of downmodulation of Ly49A correlates with increased function (37). Compared to the inhibitory Ly49A, we observe the opposite trend upon engagement of the activating receptor Ly49H, in that NK cell hyporesponsiveness correlates with increased downmodulation of the activating receptor. To our knowledge, this represents the first description that the magnitude of downmodulation of the activating receptor correlates with functional defects and suggests that, in this context, stronger activation receptor engagement results in a larger NK cell deficit.

Prior studies show that the continuous exposure of LAK cells to H60-expressing targets resulted in the downmodulation of NKG2D on the cell surface of these cells. They observed that total LAK cell lysate levels of the DAP12, DAP10, and CD3ζ proteins were decreased in these cells and the NKG2D receptor complex was altered such that NKG2D no longer appeared to associate with DAP12 or DAP10 (9, 10). The findings that continuous engagement of NKG2D altered the abundance and interaction of multiple signaling adaptor molecules differ from what we observed in our system.

Similar to continuous engagement of the NKG2D receptor with H60, engagement of Ly49H with m157 results in downmodulation of the Ly49H receptor such that the GMFI is about 10 fold lower on Ly49H^+^ NK cells from m157Tg mice compared to WT mice. However, our analysis of the total level of adaptor molecules within the NK cell did not show any significant difference in these proteins between Ly49H^+^ NK cells from WT and m157Tg mice (Figure 5A). In addition, we show that Ly49H, NK1.1, and Ly49D remain associated with their respective adaptor molecules (Figures 5B,C).

One possibility for the discrepancies between the two studies is that the NKG2D studies used LAK cells rather than primary NK cells. The high levels of IL2 as well as the length of time in which the cells grew in the cytokine could alter the phenotype and receptor complexes compared to primary NK cells used in this study. It is also possible that the interaction of NKG2D with H60 blocked the binding of the α-NKG2D antibody to the receptor, preventing the immunoprecipitation of the complex, which could account for the absence of the adaptor molecule in their pull-down assay. Finally, reduced levels of the NKG2D receptor on the cell surface caused by continuous engagement with H60 might require a larger number of these LAK cells to immunoprecipitate the adaptor molecule. In Ly49H^+^ NK cells from m157Tg mice, we demonstrate the association of DAP12 with Ly49H only upon immunoprecipitation and loading protein from drastically more cells than from WT Ly49H^+^ (Figures 5B,C). Furthermore, we show that other ITAM-associated activating receptors, including NK1.1 and Ly49D, remain associated with their adaptor molecules. This demonstrates that continuous engagement of Ly49H does not result in either the alteration of its association with DAP12 or the association of other activating receptors with their respective adaptor molecules.

A recent study identified a subset of human NK cells expressing lower levels of Syk and Zap70 kinases, which are involved in NK cell activation through ITAM signaling. The Zap70^low^/Syk^low^ NK cells, which make up a small fraction of NK cells, are functionally deficient in terms of degranulation and IFNγ production in response to K562 target cells (25). Continuous engagement of activating receptors, including CD2 and NKp46 simultaneously or NKG2D and DNAM1 simultaneously, *in vitro* could induce this phenotype, suggesting that chronic engagement of activation receptors results in lower intracellular levels of Zap70 and Syk leading to a subsequent hyporesponsive phenotype of the NK cells. Our studies demonstrate a small but statistically significant decrease in the level of Syk, but not Zap70, in Ly49H^+^ NK cells from m157Tg mice. It is possible that decreased levels of Syk but not Zap70 were seen because prior studies suggest that Syk is essential for DAP12 signaling whereas Zap70 is not (38). An interesting possibility is that Syk and Zap70 may compete for binding sites on DAP12, resulting in the initiation of different signaling pathways. The combination of decreased Syk levels and increased Zap70 levels would decrease the likelihood of Syk binding to the complex, altering the dominant signaling pathway of the receptor complex.

We also note small but statistically significant decreases in both PLCγ2 and p-PLCγ2 in Ly49H^+^ NK cells from m157Tg mice compared to WT. Although small in magnitude, such differences, particularly early in the setting of a signaling cascade, could have large impacts on functional outcomes. Furthermore, the total intracellular levels of these signaling proteins were measured, not just the protein associated with the Ly49H receptor complex, making the differences seen even more notable. Thus, alteration of Syk and PLCγ2 levels (both signaling molecules downstream of the adaptor molecules) might provide a mechanism by which continuous m157/Ly49H engagement alters other receptors' functions.

*In vivo* studies of the continuous engagement of NKG2D receptor have been performed using a transgenic model in which the human MICA protein is ubiquitously expressed (11). In these experiments using redirected killing assays, chronic NKG2D engagement resulted in impairment of killing upon stimulation through Ly49D, and to a lesser extent NK1.1, but not NKp46. The authors suggested altered expression of these activating receptors as a mechanism to explain the differences. In contrast to redirected killing, they observed increased IFNγ production by NK cells from the MICA transgenic mice compared to non-Tg mice upon stimulation with plate bound NK1.1 and NKp46 but not Ly49D. Although there appear to be altered killing and IFNγ production by NK cells, chronic engagement of NKG2D did not appear to alter B16-F10 melanoma tumor growth following subcutaneous injection *in vivo*.

There are multiple differences between our *in vivo* model of continuous engagement of activating receptors and the MICA transgenic mouse model. We observed decreased IFNγ production as well as reduced degranulation in Ly49H^+^ NK cells from m157Tg mice following cross-linking of multiple other ITAM-dependent and independent activating receptors. Furthermore, we did not find differences in the levels of other activating receptors on Ly49H^+^ NK cells from WT or m157Tg mice as was demonstrated in the MICA transgenic mice. Dissimilarities might be explained by the location and level of ligand expressed in the different transgenic mouse models. The affinity of the receptor/ligand interaction may also play a role. In addition, NKG2D expression occurs earlier in NK cell development compared to Ly49H (39–41). Finally, our study identified NK cells as NKp46^+^, CD49b^+^, and CD3^−^, while the MICA transgenic mouse study identified them as CD49b^+^, CD3^−^. All of these differences could influence how continuous receptor engagement alters NK cell function and explain the discrepancies between the two models.

Other studies suggest that continuous engagement of an activation receptor on NK cells results in defects in melanoma tumor rejection. Specifically, recombinant soluble MULT1 could stimulate rejection of subcutaneous injected melanoma cells, presumably by reversing the hyporesponsiveness of NK cells resulting from the engagement of the NK cell with an endogenously expressed NKG2D ligand on tumor-associated cells (15). We did not observe a difference in the development of lung lesions following IV melanoma injection in m157Tg vs. WT littermates (Figure 6), even though there were functional defects in Ly49H^+^ NK cells from m157Tg mice following stimulation through DNAM1, an activating receptor involved in NK cell response to melanoma cells (Figure 1B). There are several possible explanations for the differences in NK cell response in the two systems. In our model, only the Ly49H^+^ NK cells, which represents about 35% of the NK cell population, are defective. Just as the Ly49H^−^ NK cells, which do not interact with m157, exhibit no variation between WT and m157Tg in plate-bound antibody activation assays, we would expect that the Ly49H^−^ NK cells should not have any defects and be fully able to participate normally in the killing of the melanoma cells. In addition, engagement of ligand with receptor in our transgenic system was a result of the ubiquitous expression of the ligand, as opposed to the soluble MULT1 experiments, where the ligand was endogenously expressed and not transgenic. Although we did not see an *in vivo* response to the administration of melanoma cells, we observed that continuous Ly49H engagement alters the *in vivo* NK cell response to target cells that have downregulated MHC class I (missing- self).

NK cell responses to tumor cells result from interactions that take place between ligands expressed on cells within the tumor environment and the receptors expressed on the NK cells, such as inhibitory receptors that can bind MHC class I molecules and activating receptors that can bind stress-induced ligands. Tumors cells can decrease the expression of MHC class I on their surface in order to evade the adaptive immune system (42). In doing so, tumors become more susceptible to killing by NK cells due to loss of engagement with inhibitory receptors. We demonstrate that not only does continuous engagement of an activating receptor result in defects in IFNγ and degranulation when the NK cell is stimulated through other activating receptors *in vitro*, but there is also a defect in killing cells that have lost expression of MHC class I *in vivo*. This could have a major impact on the ability of NK cells to kill tumor cells in the tumor microenvironment.

A study analyzing peritoneal fluid NK cells from patients with ovarian cancer noted that tumor-associated NK cells expressed lower levels of several receptors, including DNAM1, compared to NK cells from the peripheral blood, and these peritoneal NK cells were hyporesponsive in killing HLA class I deficient targets. Furthermore, incubation of NK cells with ovarian cancer cells expressing the DNAM1 ligand CD155 resulted in downmodulation of DNAM1 on the NK cells (43). This suggests that chronic engagement of an activating receptor expressed on tumor-associated NK cells may hamper immune surveillance and promote tumor growth. In fact, reduced surface expression of DNAM1 and NKG2D has been associated with defective NK cell cytotoxicity and cytokine production in patients with various types of advanced cancer (44–47). Furthermore, studies show that glioblastoma cells produce lactate dehydrogenase 5, which can induce the expression of NKG2D ligands on both tumor-infiltrating myeloid cells and circulating monocytes. This results in the chronic engagement of the NKG2D receptor on NK cells and downmodulation of the NKG2D receptor (48). It is possible that upregulation of NKG2D ligands within the tumor environment results in continuous engagement of the activating receptor NKG2D on the intra-tumor NK cells and their subsequent hyporesponsiveness. Blocking this type of continuous engagement, as suggested by the soluble MULT1 experiments, may prevent NK cell hyporesponsiveness (15, 49, 50).

Of note, ITAM bearing adaptor molecules appear to be capable of mediating inhibitory signals in myeloid derived cells (51, 52). In fact, the DAP12 adaptor molecule, associated with multiple NK cell activating receptors including Ly49H, can act to mediate inhibitory signals in osteoclast and macrophages (51, 53, 54). Whether the ITAM motif is activating or inhibitory might depend on the strength of the interaction of the receptor with the ligand, the cell type that expresses the ligand, as well as the length of time the interaction takes place. The potential mechanisms by which it can alter signaling through other activating receptors include the sequestration of a critical component involved in signaling by the other receptor as well as possibly the recruitment of a phosphatase to the complex which dephosphorylates the signaling cascade of the other receptor (53). It is possible that similar changes are taking place in NK cells following continuous engagement of the activating receptor.

The continuous engagement of the Ly49H receptor appears to result in modification of the downstream signaling cascade that alters effector response to stimulation by other ITAM-dependent and independent receptors. In addition, there are defects in the NK cell ability to respond to missing-self. These mechanisms likely play a role in how NK cells respond to both tumor cells and virally infected cells, two scenarios in which downmodulation of MHC class I takes place. This makes understanding the mechanisms by which continuous activation receptor engagement results in defective NK cell function an important prerequisite for the development of potential new therapies to make NK cells more efficient at eliminating tumor cells.

## Data Availability

All datasets generated for this study are included in the manuscript/Supplementary Files.

## Ethics Statement

All animals received humane care according to the criteria outlined in the Guide for the Care and Use of Laboratory Animals prepared by the National Academy of Sciences and published by the National Institutes of Health (NIH publication 86-23 revised 1985). The Animal Studies Committee at Washington University (St. Louis, MO) approved all animal studies.

## Author Contributions

PK, JG, LY, and SP performed the experiments. PK, JG, SP, and ST designed the experiments and analyzed the data. PK and ST wrote the manuscript. All authors have read and approved the manuscript.

### Conflict of Interest Statement

The authors declare that the research was conducted in the absence of any commercial or financial relationships that could be construed as a potential conflict of interest.
