# Supplementary material for: Chronic Ly49H Receptor Engagement in vivo Decreases NK Cell Response to Stimulation Through ITAM-Dependent and Independent Pathways Both in vitro and in vivo
Source: Front Immunol. 2019 Jul 23;10:1692. doi: 10.3389/fimmu.2019.01692 (PMC6664057; doi:10.3389/fimmu.2019.01692)
Supplement: Supplementary file 1 [file Presentation_1.pptx]

## Slide 1
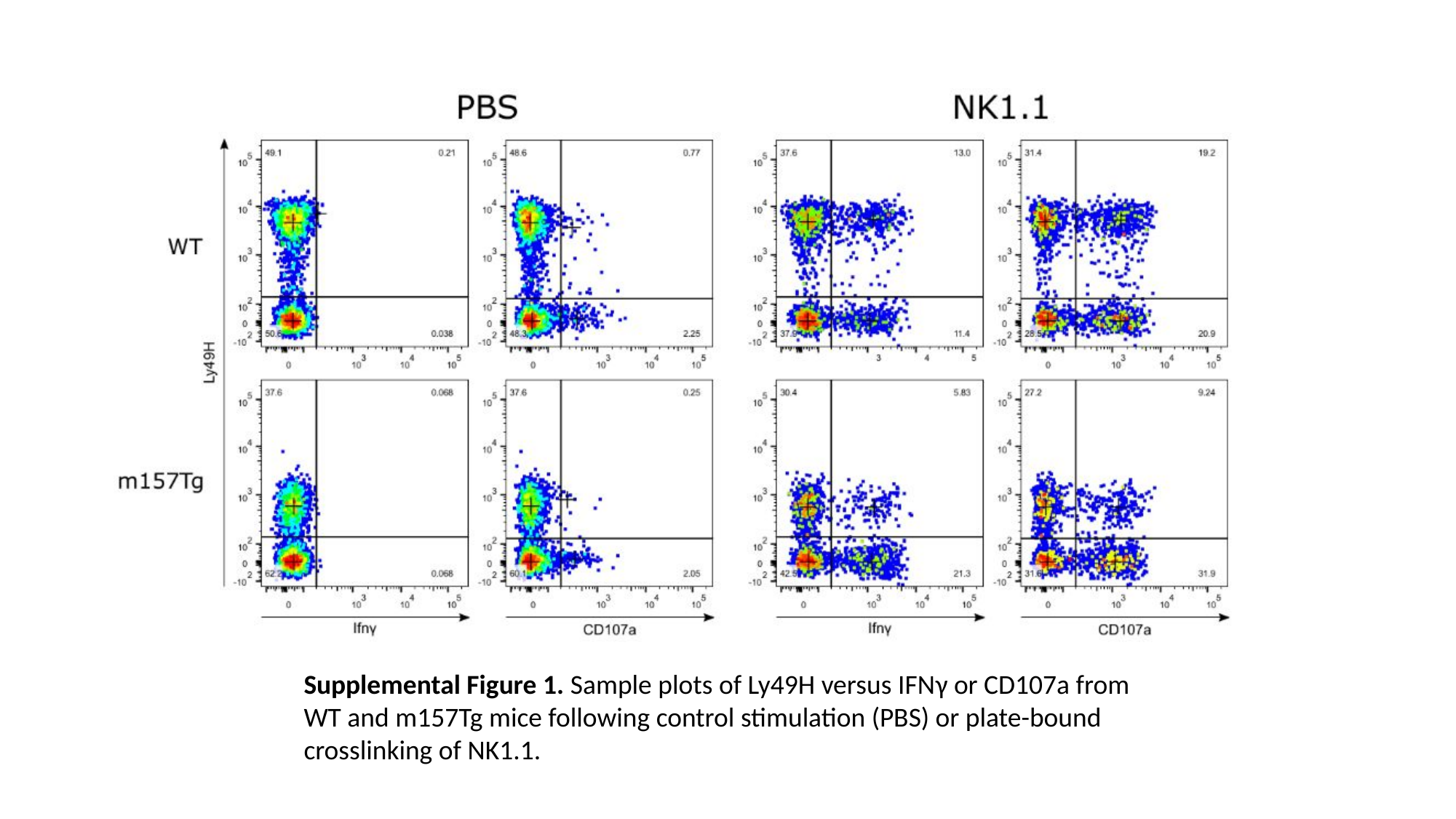

Supplemental Figure 1. Sample plots of Ly49H versus IFNγ or CD107a from WT and m157Tg mice following control stimulation (PBS) or plate-bound crosslinking of NK1.1.

## Slide 2
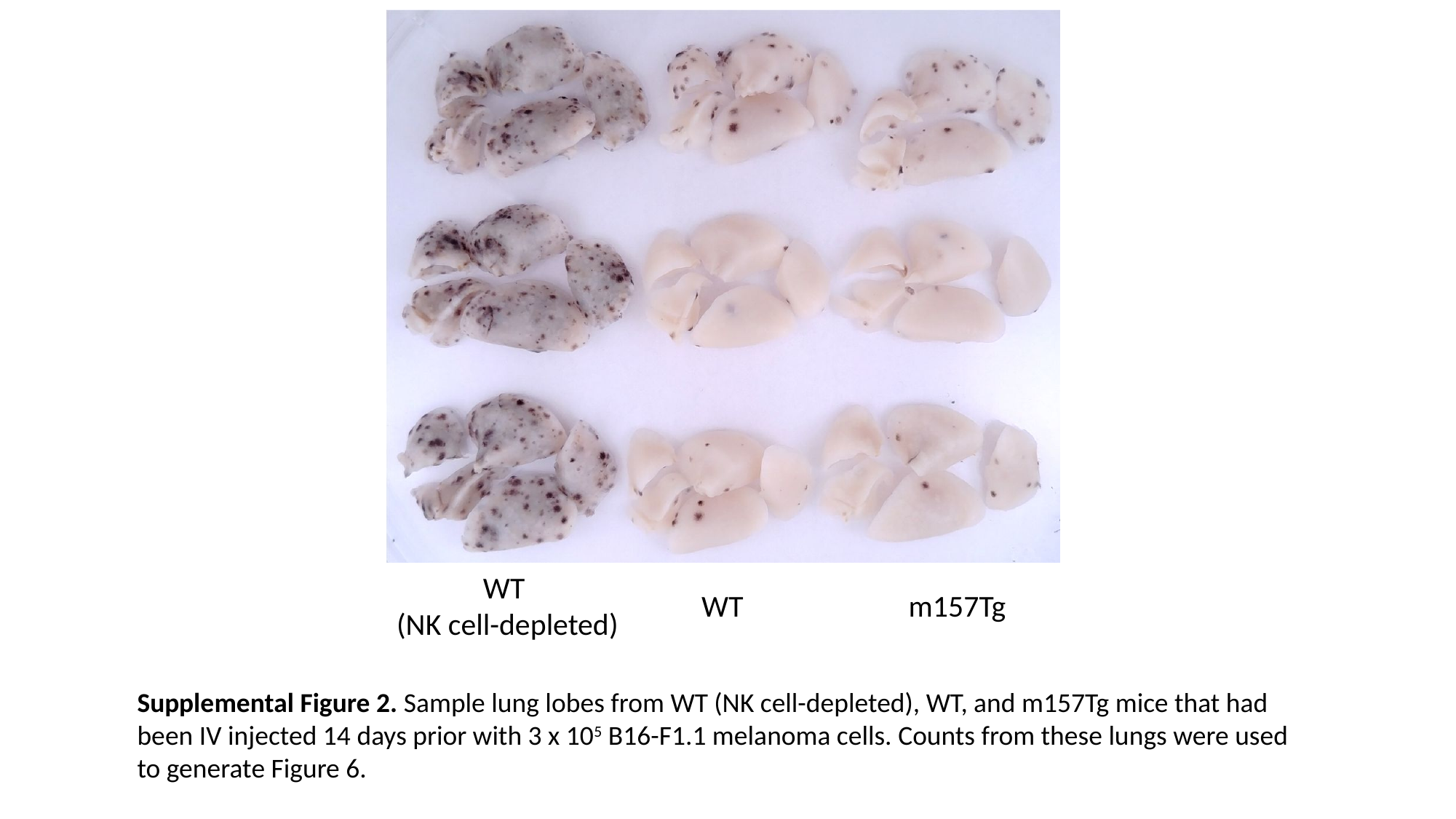

WT
(NK cell-depleted)
WT
 m157Tg
Supplemental Figure 2. Sample lung lobes from WT (NK cell-depleted), WT, and m157Tg mice that had been IV injected 14 days prior with 3 x 10^5^ B16-F1.1 melanoma cells. Counts from these lungs were used to generate Figure 6.
